# The Ratio of IP10 to IL-8 in Plasma Reflects and Predicts the Response of Patients With Lung Cancer to Anti-PD-1 Immunotherapy Combined With Chemotherapy

**DOI:** 10.3389/fimmu.2021.665147

**Published:** 2021-04-12

**Authors:** Liangliang Wu, Shengzhi Xie, Lingxiong Wang, Jinfeng Li, Lu Han, Boyu Qin, Guoqing Zhang, Qiyan Wu, Wenjuan Gao, Lijun Zhang, Huafeng Wei, Tianyi Liu, Shunchang Jiao

**Affiliations:** ^1^ Institute of oncology, The Fifth Medical Centre, Chinese PLA General Hospital, Beijing, China; ^2^ Department of Oncology, The Fifth Medical Centre, Chinese PLA General Hospital, Beijing, China

**Keywords:** combination therapy, immunotherapy, cytokines, IP10, IL-8

## Abstract

Antibodies against checkpoint inhibitors such as anti-programmed cell death protein 1 (PD-1) and its ligand anti-programmed death ligand 1 (PD-L1) have shown clinical efficacy in the treatment of multiple cancers. However, there are only a few studies on biomarkers for these targeted immunotherapies, especially in peripheral blood. We first studied the role of interferon-induced protein-10 (IP10) combined with interleukin-8 (IL-8) in peripheral blood as a biomarker of immune-combined chemotherapy for lung cancer and multiple cancers. We used the high-throughput cytokine detection platform and performed bioinformatics analysis of blood samples from 67 patients with lung cancer and 24 with multiple cancers. We selected the ratio of IP-10 to IL-8 (S2/S0, ratio of changes at 10–12 weeks after treatment to baseline) to predict the response to immunotherapy combined with chemotherapy and evaluate the survival of lung cancer patients and mixed cancer patients. In patients treated with the combination therapy, the specificity and sensitivity of IL-8 and IP10 together as predictors were improved compared with those of IL-8 and IP10 alone. Our conclusion was verified in not only lung cancer but also multiple cancer research cohorts. We then further validated the predictive effect of biomarkers in different histologic types of NSCLC and chemotherapy combined with different PD-1 drug groups. Subsequent validation should be conducted with a larger number of patients. The proposed marker IP10 (S2/S0)/IL-8 (S2/S0), as a predictive immunotherapy biomarker, has broad prospects for future clinical applications in treating patients with multiple intractable neoplasms.

## Introduction

The use of immunotherapy for malignancy was first proposed in 1891 by Dr. William Coley (1). With the molecular identification of tumor antigens (2), immunotherapy has been widely used in multiple cancer treatment strategies. Tumors express programmed death ligand 1 (PD-L1), which interacts with programmed cell death 1 (PD-1) on T cells to negatively regulate the immune response and evade host immune surveillance (3). Immune checkpoint inhibitors (ICIs) targeting PD-1 and its ligand (PD-L1) have substantially changed the landscape of cancer treatment over the last 5 years. ICIs induce durable responses and prolong the survival of patients with advanced cancers by blocking the activity of the co-inhibitory receptor, key to the T-cell immune response, and consequently promoting T-cell-mediated antitumor activity (3, 4). Drugs targeting this negative immune regulatory pathway, including the anti-PD-1 monoclonal antibodies (mAbs) nivolumab (5), pembrolizumab (6), and cemiplimab (7) and the anti-PD-L1 mAbs atezolizumab (8), avelumab (9), and durvalumab (10) have been approved for the treatment of various cancer types. These agents have now become first-line and adjuvant therapies, particularly for the treatment of lung cancer. However, only some patients with cancer are responsive to ICI treatment for most cancer types (3, 11, 12). Recently, it has been reported that immunotherapy combined with chemotherapy results in a longer progression-free survival (PFS) and overall survival (OS) for patients (13, 14). Considering the high cost and potential immune-related adverse effects, it is of critical importance to identify and develop reliable, highly specific/sensitive, and easy-to-use biomarkers to predict the clinical outcomes of ICI treatment.

Predictive biomarkers of the response to anti-PD-1/PD-L1 ICIs have been widely investigated. Studies have demonstrated that the expression of PD-L1 in tumor tissues and the infiltration of hematopoietic cells within the tumor are correlated with the response to anti-PD1 immunotherapy (12, 15, 16). Microsatellite instability has been considered a common response biomarker to monitor a variety of solid tumors treated with pembrolizumab, which was approved by the FDA (17). Furthermore, recently reported peripheral circulation biomarkers include circulating immune cells and peripheral blood cytokines. The biological characteristics of peripheral blood CD8 T cells are associated with clinical responses in patients treated with nivolumab for non-small cell lung cancer (18), and the frequency of activated monocyte and neutrophil counts have been shown to predict PFS and OS in patients treated with ICIs (19, 20). Cytokines are immune regulators that promote the recruitment of immune cells into the tumor microenvironment (21–23). Peripheral circulating cytokines have been reported to predict immune-related toxicity in patients with melanoma treated with PD-1 (24). In a study of 29 patients with melanoma treated with nivolumab or pembrolizumab, the serum IL-8 level was strongly associated with therapy response, which was validated in 19 patients with Non-small cell lung cancer (NSCLC) treated with these agents (25). Chemokines play a vital role in T cell infiltration, and the interaction between CXCR3 and its ligands CXCL9, CXCL10, and CXCL11 plays a key role in T cell distribution and migration. Chow et al. recently demonstrated that the levels of circulating CXCL9 and CXCL10 in patients with melanoma might serve as biomarkers for treatment with PD1 (26). However, owing to the complicated nature of the tumor immune microenvironment, the predictive value of these single markers is insufficient for clinical application. Previously, it was found that IL-8 and IP10 alone are associated with tumor burden and immunotherapy efficacy, but their combination as biomarkers has not been addressed.

In this study, through a high-throughput cytokine detection platform, the content of cytokines in peripheral blood plasma of 91 enrolled patients at three time points (S0, collected before therapy initiation; S1, weeks 4–6 after therapy initiation; S2, weeks 10–12 after therapy) before and after treatment was detected. WE selected IP10 combined with IL-8 cytokines (S2/S0, ratio of changes at 10–12 weeks after treatment to baseline) to predict the response to immunotherapy combined with chemotherapy and evaluate the survival of 67 patients with lung cancer and 24 patients with mixed cancer. We proposed the combination of IL-8 and IP10 as a biomarker for immunotherapy in patients with multiple cancers, especially lung cancer. This marker improved the specificity and sensitivity as a predictor compared to those with IL-8 and IP10 alone. The proposed model has high clinical application value as an effective biomarker to screen patients who would benefit from anti-PD-1 therapy and to determine the survival of patients with cancer.

## Materials and Methods

### Study Design and Participants

From March 2016 to March 2019, 91 patients treated with PD-1–PD-L1 pathway blockade immunotherapy in the Department of Oncology of Chinese PLA General Hospital were included in this study. The discovery cohort comprised 67 patients with lung cancer and the validation cohort comprised 24 patients with multiple cancers. The recruited patients received chemotherapy including albumin taxol, cis-platinum, carboplatin, gemcitabine, docetaxel, and docetaxel combined with immunotherapy such as nivolumab (dosage, 3 mg/kg) and pembrolizumab (dosage, 2 mg/kg). The immune response effects were defined via two different approaches as follows: clinical benefit (CB) and no clinical benefit (NCB); patients with cancer were defined as those with PFS >180 days and not >180 days, respectively; response (R) and no response (NR) were defined based on the remission degree of tumor lesions as complete response (CR), partial response (PR), or stable disease (SD) and progressive disease (PD). This study was approved by the ethics committee of Chinese PLA General Hospital; the informed consent of all patients participating in this study was obtained.

### Plasma Samples

Peripheral blood samples from patients were collected prospectively at baseline (S0, collected before therapy initiation) and at regular intervals during therapy, including early (S1; weeks 4–6 after therapy) and late (S2; weeks 10–12 after therapy). Whole blood was collected in vessels containing EDTA (BD) and processed within 4 h of collection. At room temperature, the blood samples were centrifuged at 1500 rpm (800 × *g*) for 15 min to collect plasma, followed by a second centrifugation at 4100 rpm (1600 × *g*) for 10 min for further plasma clearance. The clarified plasma samples were frozen in a −80°C refrigerator for further testing.

### Multiplex Plasma Cytokine Analysis

Plasma samples were obtained using a commercial kit (Bio-Plex Pro Human Cytokine Grp I Panel 27-Plex; Bio-Rad, USA) on the Bio-Plex 200 System. The detected cytokines included Th1 type cytokines (IL-2, IL-7, IL-12p70, IL-15, IFN-γ, and IP-10), Th2 (IL-4, IL-5, and IL-13), proinflammatory cytokines (IL-1β, IL-1RA, IL-6, IL-8, IL-17A, and TNF-α), an immunoregulatory cytokine (IL-10), growth factors (FGF-basic, VEGF, G-CSF, and GM-CSF), and several other chemokines (IL-9, MIP-1α, MIP-1β, MCP-1, RANTES, Eotaxin, and PDGF-BB). Briefly, a gradient dilution standard was prepared according to the specification, and each standard was thoroughly mixed during the preparation process. The diluted plasma was incubated in plates containing magnetic antibody-coupled beads and incubated under shock for 30 min. Thereafter, the sample was washed and incubated with the secondary antibodies, as described previously herein. Streptavidin-PE was added to the sample at the end of incubation and washing. Bio-plex Manager 5.0 Software was used to obtain the raw data. The concentrations of 27 cytokines were determined using standard curves. Quality control standards were strictly followed during the experiment.

### Statistical Analysis

Data are reported as the median and range. A Wilcoxon rank sum test was used to analyze significant differences in single factors between the two groups. The joint factor screening algorithm mainly included factors through iteration, to ensure that the logistic regression model fitted by each included factor can optimize the area under curve (AUC) of both training and validation sets. After including each factor, the coefficient significance of the factors was statistically significant (default P < 0.05), and the increase in the evaluation function was greater than k (default k = 2). The effect of treatment on OS or PFS was analyzed using Kaplan–Meier curves. A logarithmic rank test was used to compare the survival curves between the two groups. R software (version 3.6.0) and Perl software (version 5.2.2) were used for data management and analyses. The significance level for all results was set at P < 0.05.

## Results

### Cytokines Are Associated With the Effectiveness of Chemo-Combined Immunotherapy Against Lung Cancer

Whether cytokines can act as biomarkers that predict the efficacy of human PD-1 blocking therapy is still controversial. In this study, we determined the cytokine levels in 67 patients with lung cancer (discovery cohort) and verified them in 24 patients with multiple cancers (validation cohort). Data of 27 cytokines from 91 patients were obtained in three periods, specifically 0, 4–6, and 10–12 weeks after immunotherapy combined with chemotherapy, with the corresponding periods labeled S0, S1, and S2 samples. Patient characteristics are listed in **Table 1**.

**Table 1:** Patient baseline characteristics.

Characteristics	Discovery Cohort	Validation Cohort
	Lung cancer(N=67)	Multiple cancer types(N=24)
Median age	56.7(34-87)	58.3 (34-80)
Sex		
Male	47(70.1)	12 (50.0)
Female	20 (29.9)	12 (50.0)
ECOG		
0	18 (26.8)	0 (0.0)
1	33 (49.3)	19 (79.2)
≥2	16 (23.9)	5 (20.8)
Somking Status		
Never	30 (44.8)	19 (79.2)
Former	23 (34.3)	5 (20.8)
Current	14 (20.9)	0 (0.0)
Stage		
II	2 (3.0)	0 (0.0)
III	13 (19.4)	1(4.2)
IV	52 (77.6)	23 (95.8)
Tumor Histology		
Adenocarcinoma	36 (53.7)	16 (66.7)
Squamous	21 (31.4)	3 (12.5)
Other	10 (14.9)	5 (20.8)
Mutation		
BRAF or NRAS	1 (1.5)	NA
KRAS	6 (9.0)	NA
EGFR	14 (20.9)	1(4.2)
ALK	2 (3.0)	NA
Treatment		
Nivolumab	26 (38.8)	13 (54.2)
Pembrolizumab	38 (56.7)	9 37.5)
others	3 (4.5)	2 (8.3)
No. of prior systematic therapies		
0	15 (22.4)	3 (12.5)
1	28 (41.8)	14 (58.3)
2	10 14.9)	4 (16.7)
≥3 Confirmed ORR, n (%) Best overall response Complete Response Partial Response Stable Disease Progressive Disease	14 (20.9) 19 (28.35) 0 19 (28.35) 29 (43.3) 19 (28.35)	3 (12.5) 7 (29.2) 0 7 (29.2) 8 (33.3) 9 (37.5)

The 24 patients in the validation group included 11 for gastric cancer, 4 for esophageal cancer, 4 for ovarian cancer, 2 for breast cancer, 1 for colon cancer, 1 for liver cancer and 1 for melanoma.

**Figure 1:**
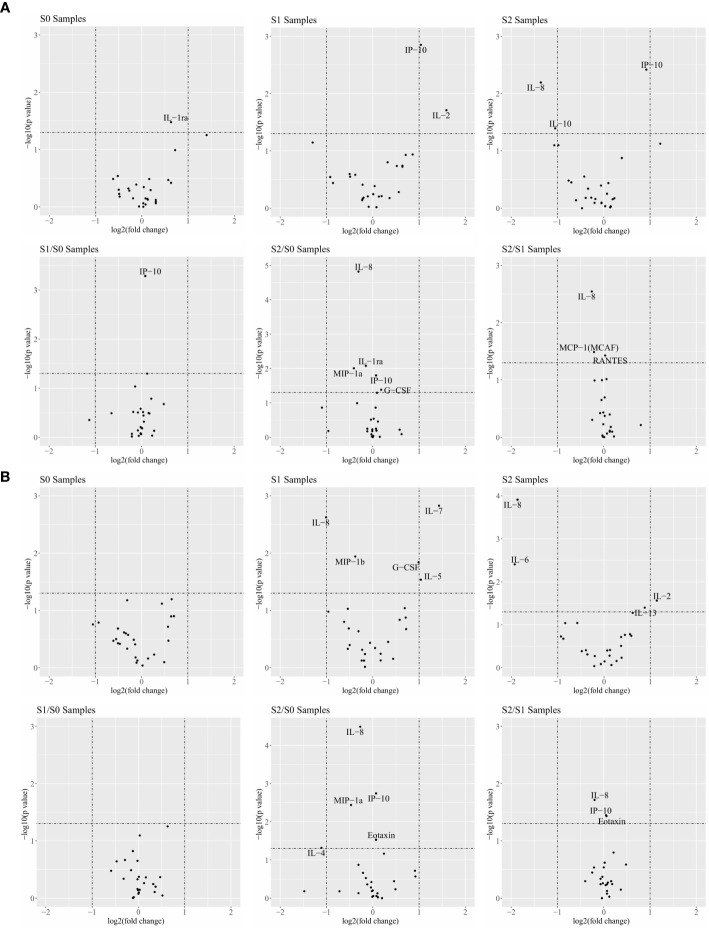
Cytokines are associated with the response of combination therapy in responder (R)/non-responder (NR) patients and patients with clinical benefit/no clinical benefit (NCB). **(A)** Comparison of 27 cytokines in R patients and NR patients at different time points and different ratios in the volcano map. **(B)** Comparison of 27 cytokines in CB and NCB patients at different time points and different ratios in the volcano map. Cytokines with a log_2_ |fold-change| greater than 1.0 and a −log10 (P-value) greater than 1.3 were considered significant.

In the responder and non-responder classification, the volcano plot analysis of 27 cytokines was conducted according to the fold-change and P-values. Cytokines with log_2_ (fold change) greater than 1.0 and –log_10_ (P value) greater than 1.3 were considered significant. IL-2 and IP-10 of the responders in the S1 samples were significantly higher than those of the non-responders. However, the S2 sample respondents had lower levels of IL-8 and IL-10, but there was no statistical difference between the R (response) and NR (no-response) groups in the S0, S1/S0, S2/S0 and S2/S1 samples (**Figure 1A** and **Supplementary Table 1**). The same analytical strategy was used in the clinical benefit and no clinical benefit groups. The volcano plot analysis of these cytokines demonstrated that IL-7 and IL-5 in the S1 samples and IL-2 in the S2 samples were significantly elevated. However, the levels of IL-8, IL-6, and IL-4 in the S1, S2, and S2/S0 samples were lower in the clinical benefit patients. There was no difference in cytokines the other groups between CB (clinical benefit) and NCB (no-clinical benefit) comparisons (**Figure 1B** and **Supplementary Table 1**).

The result suggest the following: Th1 cytokines (IL-2 and IP10) were significantly increased and Th2 cytokines were significantly decreased in patients who responded and benefited after clinical treatment, and proinflammatory cytokines and external immune regulation (IL-6 and IL-10) were significantly reduced. IL-10 plays an important role in immune regulation, previous studies have also shown that IL-10 can inhibit the antigen-presenting function of macrophages and inhibit the production of IFN-γ by Th1 cells, IL-10 is involved in suppressing the Th1 immune response *in vivo* (27, 28).

### Combination of Plasma Cytokines Acts as a Predictive Biomarker

In the previous section, we highlighted several clinically significant indicators through statistical analysis in different analysis groups. We then used stepwise discriminant analysis to elucidate superior biomarkers based on cytokine combinations. A differential analysis was conducted with 162 items (27 cytokines × 3 time points + 27 cytokines × 3 ratios (fold-change; S1/S0, S2/S0, and S2/S1) (**Supplementary Table 2**). By determining the logarithm base 2 value of each factor, 17 and 15 factors with significant differences were found in the CB/NCB and R/NR groups, respectively. The stepwise Akaike’s information criterion regression analysis revealed that cytokine combinations I and II were the most predictive of the S0+S1 and S0+S1+S2 samples in the R/NR (**Supplementary Table 3**) and CB/NCB groups (**Supplementary Table 4**), respectively. The two combinations mainly included IL-2, IL-4, IL-6, IL-7, IL-8, IP10, and MCP-1. Each combination is listed from top to bottom according to the importance of cytokines.

**Figure 2:**
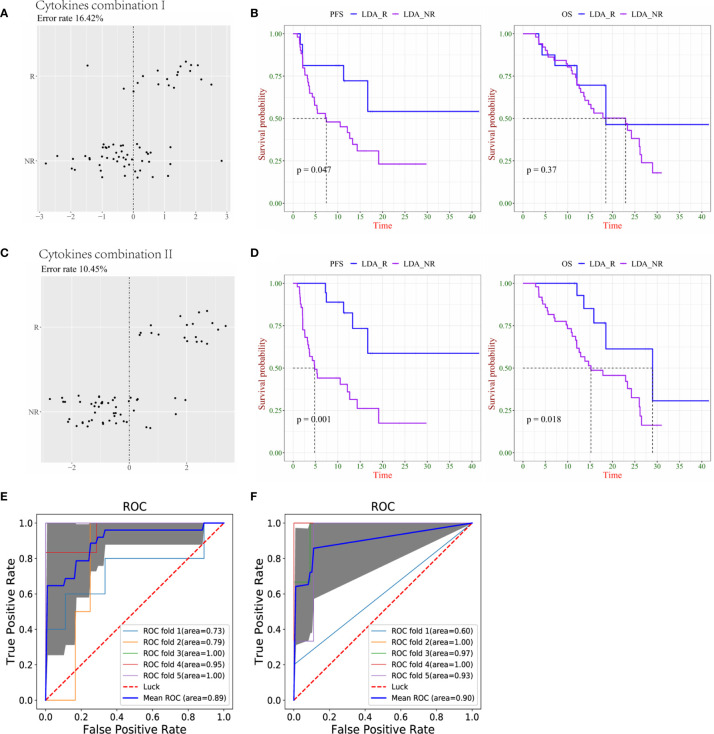
Combination of cytokines could predict survival more precisely in responder (R) and non-responder (NR) lung cancer patients. **(A)** The accuracy of cytokine combination I was evaluated by linear discriminant analysis (LDA). **(B)** Kaplan–Meier plots of progression-free survival (PFS, left) and overall survival (OS, right) of LDA-R and LDA-NR based on cytokine combination I. **(C)** The accuracy of cytokine combination II was evaluated by LDA. **(D)** Kaplan–Meier plots of PFS (left) and OS (right) of LDA-R and LDA-NR based on cytokine combination II. **(E)** The ROC curve of 5-fold cross-validation for cytokine combination I **(F)** The ROC curve of 5-fold cross-validation for cytokine combination II.

We performed a linear discriminant analysis (LDA) and used cytokine combination I to distinguish patients who responded to treatment from those who did not for the S0+S1 sample with an error rate of 16.42%. To verify the reliability of the obtained cytokine combination 1, according to the LDA cutoff value, 67 samples were divided into LDA-R or LDA-NR (**Figure 2A**). For PFS, there was a statistically significant difference between LDA-R and LDA-NR, whereas there was no statistically significant difference in the OS (**Figure 2B**). Furthermore, we found that cytokine combination II had a lower error rate than combination I in differentiating R and NR groups; the error rate was 10.45% (**Figure 2C**). There were significant differences in PFS and OS between LDA-R and LDA-NR (**Figure 2D**). To use the discriminant model to calculate the AUC of cytokine combinations, logistic regression was used to perform five-fold cross validation, using the cross-validation method. For data queue grouping, we used 80% of the data to build the model and 20% to validate; AUC was used to evaluate the performance of the model. The AUC of cytokine combinations I and II was 0.89 and 0.90, respectively, indicating that combination II better distinguished R and NR patients (**Figures 2E, F** **)**.

**Figure 3:**
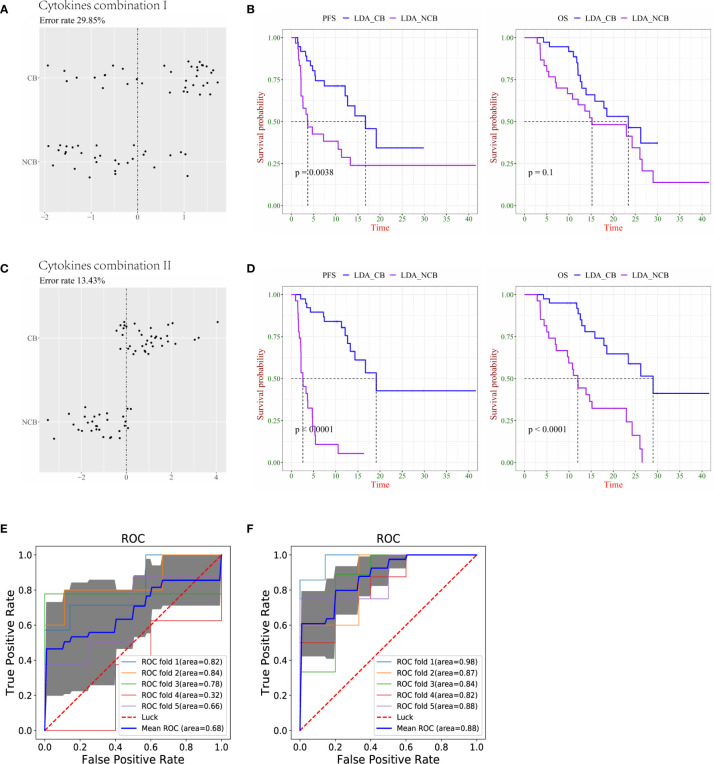
Combination of cytokines predicts survival more precisely in lung cancer patients with clinical benefit (CB) and no clinical benefit (NCB). **(A)** The accuracy of cytokine combination I was evaluated using the linear discriminant analysis (LDA). **(B)** Kaplan–Meier plots of progression-free survival (PFS, left) and overall survival (OS, right) of LDA-CB and LDA-NCB based on cytokine combination I. **(C)** The accuracy of cytokine combination II was evaluated using the LDA. **(D)** Kaplan–Meier plots of PFS (left) and OS (right) of LDA-CB and LDA-NCB based on cytokine combination II. **(E)** The ROC curve of 5-fold cross-validation for cytokine combination I. **(F)** The ROC curve of 5-fold cross-validation for cytokine combination II.

In terms of clinical benefit versus no clinical benefit classifications, the LDA demonstrated that cytokine combination I distinguished between the CB and NCB groups in the S0+S1 sample with an error rate of 29.85% (**Figure 3A**). Similar to the results of the N/NR group, there was a significant difference in PFS between LDA-CB and LDA-NCB, but no statistical difference in OS (**Figure 3B**). Similarly, combination II had a lower error rate (13.43%) than combination I (**Figure 3C**), and both resulted in significant differences in PFS and OS between LDA-CB and LDA-NCB (**Figure 3D**). The AUC calculated by 5-fold cross-validation values was 0.68 and 0.88 for cytokine combinations I and II, respectively, suggesting that combination II better discriminated CB from NCB groups (**Figures 3E, F** **)**.

These results were based on the analysis and presentation of lung cancer data, Subsequently, we analyzed the cases of multiple cancers, and only the PFS and OS of cytokine combination 2 between LDA-CB and LDA-NCB in the CB/NCB group showed a statistical difference, whereas there was no statistical difference in the other groups (**Supplemental Figures 1A–D**). Overall, cytokine combinations I and II can be used as predictive biomarkers for immunotherapy combined with chemotherapy, especially in the lung cancer group. As a predictor, combination II presented a lower prediction error rate and higher specificity and sensitivity in the R/NR and CB/NCB groups. It was also associated with better clinical outcomes. It is noteworthy that IL-8 (S2/S0) and IP10 (S2/S0) were included in combination II of the R/NR and CB/NCB groups.

### Optimum Factor Combination Calculated Using a Novel Regression Approach

Here, we propose a new algorithm to select a combination of factors, by optimizing not only the fitness of the training set but also the generalization of the validation set. Using this algorithm, IL8 in the S2/S0 period and IP10 in the S2/S0 period were selected as the combination factors for the R/NR and CB/NCB grouping models (**Table 2**). The fitting coefficients of the two groups were calculated using the logistic regression model (**Supplementary Table 5**). We then transformed the logistic regression model (**Supplementary Table 6**), and as the coefficient was from the training set, we could further transform the two cytokines into one factor IP10 (S2/S0)/IL8 (S2/S0) as a biomarker of the clinical response effect. There was no significant difference in the AUC between this biomarker and the logistic regression model in the lung cancer and multiple cancer groups (**Table 3**). Therefore, we used this indicator in the subsequent analysis and verification.

**Table 2:** The best predictive combination of cytokines selected by the new algorithm.

Group	Cytokines	AUC in training set	AUC in validation set	Evaluation Function	Coefficient Significance P
CB/NCB	IP10 (S2/S0)	73.6	88.89	28.64	0.00311
	IP10 (S2/S0)+IL8 (S2/S0)	87.93	96.53	12.56	0.001937, 0.000714
R/NR	IL8 (S2/S0)	84.54	72.69	31.38	0.000426
	IL8 (S2/S0)+IP10 (S2/S0)	89.25	89.08	15.32	0.000562, 0.022791

**Table 3:** Comparison of AUC between IP10/IL8 (S2/S0) and logistic regression model in different sets.

Model	Group	AUC (lung cancer)	AUC (Multiple cancers)
Logistic regression	CB/NCB	0.8793	0.9653
	R/NR	0.8925	0.8908
IP10/IL8 (S2/S0)	CB/NCB	0.8784	0.9653
	R/NR	0.8887	0.8992

### Combination of New Factors Can Predict Survival More Precisely in Patients With Lung Cancer and Multiple Cancers

**Figure 4:**
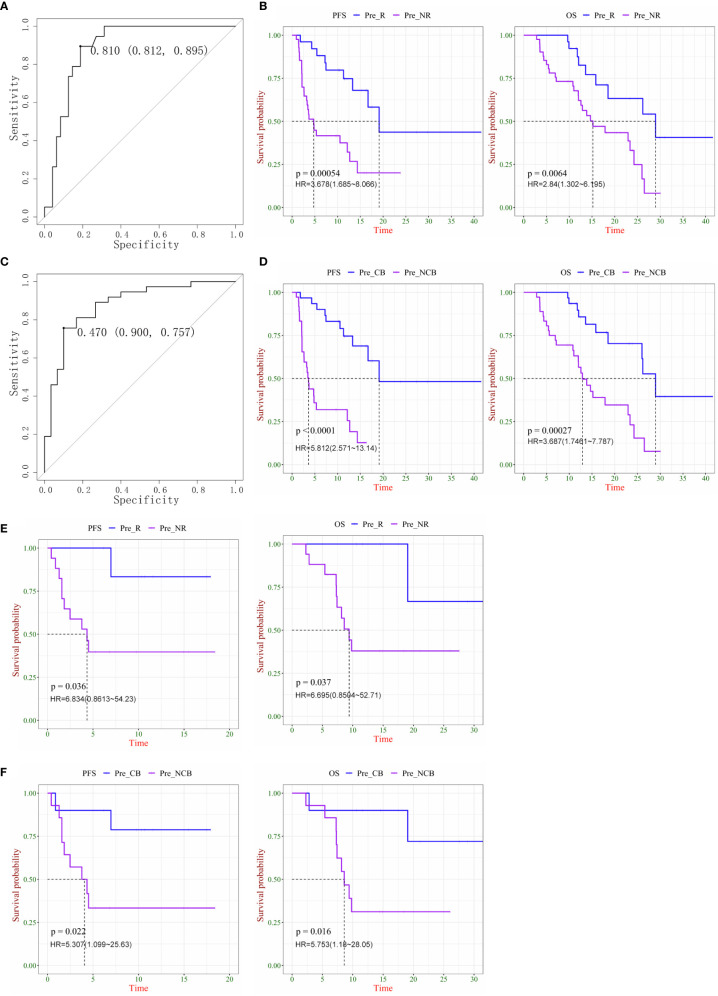
Plasma ratio of IP-10 to IL-8 was associated is progression-free survival (PFS) and overall survival (OS) in chemo-immunotherapy patients with cancer. **(A)** ROC curve as the threshold of the immune response in the R/NR groups. **(B)** Kaplan–Meier survival curves of PFS (left) and OS (right) in R and NR patients with lung cancer. **(C)** ROC curve as the threshold of the immune response in CB and NCB patients with lung cancer. **(D)** Kaplan–Meier survival curves of PFS (left) and OS (right) in CB and NCB patients with lung cancer. **(E)** Kaplan–Meier survival curves of PFS (left) and OS (right) in R and NR patients with multiple cancers. **(F)** Kaplan–Meier survival curves of PFS (left) and OS (right) in CB and NCB patients with multiple cancers. R, responder; NR, non-responder; CB, clinical benefit; NCB, no clinical benefit; HR, hazard ratio.

After obtaining the combination factor IP10/IL8 (S2/S0), 67 patients were used as the training set to train the logistic regression model of CB/NCB and R/NR. The ROC curves displaying the maximum sensitivity and specificity were calculated as the threshold of the immune response, as R/NR = 0.81 (**Figure 4A**) and CB/NCB = 0.47 (**Figure 4C**); survival curves were constructed using the predicted values, IP10/IL-8 (S2/S0) was used as a predictive marker, the Cut-off value of R/NR and CB/NCB was 1.753 and 1.385 respectively. The model threshold was used to predict the immune response effect of R/NR and CB/NCB. IP10/IL-8 (S2/S0) was used as a predictor of both clinical response and clinical benefit for patients receiving chemotherapy combined with immunotherapy for lung cancer(n=67). There were significant differences in PFS and OS (**Figures 4B, D** **)**. IP10/IL-8 (S2/S0) was also validated as biological predictor in multiple cancers(n=24), and it had statistical significance in distinguishing CB/NCB and R/NR and in judging the prognosis (PFS and OS) of patients (**Figures 4E, F** **)**.

With these results, we obtained a new prediction marker, IP10/IL-8 (S2/S0); for the lung cancer group or mixed carcinoma queue, it showed a better prediction effect. This indicator was more clinically significant than the cytokine combination I and combination II. IP10 and IL-8 play an important role in tumor promotion and inhibition, combining these two factors as biomarkers has more clinical significance, and an increased IP10/IL-8 (S2/S0) ratio indicates that patients can benefit more from the treatment.

### Further Analysis of Biomarkers in Different Pathological Types of NSCLC and PD-1 Drugs

**Figure 5:**
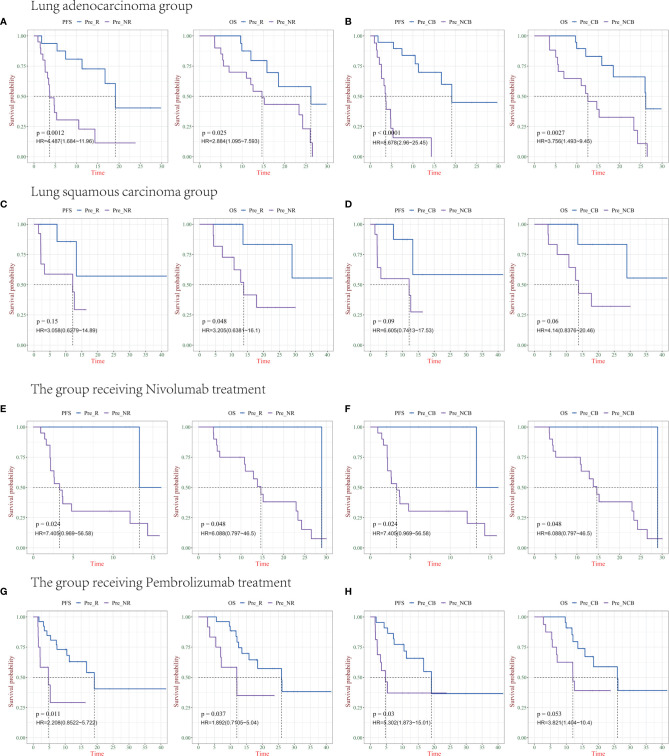
Survival analysis of IP10/IL-8 (S2/S0) in different pathological types of Non-small cell lung cancer (NSCLC) and PD-1 drugs. **(A, B)** progression-free survival (PFS) and overall survival (OS) in patients with lung adenocarcinoma in the R/NR (left) and CB/NCB (right) groups (n = 36). **(C, D)** PFS and OS in patients with R/NR (left) and CB/NCB (right) lung squamous cell carcinoma (n = 21). **(E, F)** PFS and OS in patients with receiving chemotherapy combined with nivolumab in the R/NR (left) and CB/NCB (right) groups (n = 26). **(G, H)** PFS and OS in patients receiving chemotherapy combined with pembrolizumab in the R/NR (left) and CB/NCB (right) groups (n = 38). R, responder; NR, non-responder; CB, clinical benefit; NCB, no clinical benefit; HR, hazard ratio.

In the 67 patients we previously analyzed, including 57 cases of non-small cell lung cancer, we used the previously obtained indicators to validate two groups of tumors with different pathological types. This validation group included 21 cases of squamous cell carcinoma and 36 cases of adenocarcinoma. The PFS and OS of the patients were analyzed according to different clinical efficacies. There were statistically significant differences in PFS and OS among groups with clinical response or non-response for lung adenocarcinoma (P = 0.0012, P = 0.025), and there were also statistically significant differences in PFS and OS among groups with clinical benefit or non-benefit (P<0.0001, P = 0.0027) (**Figures 5A, B** **)**. Among patients with lung squamous cell carcinoma, only clinical benefit and non-benefit groups had statistically significant overall survival (P = 0.048), and there was no statistical difference in other groups (**Figures 5C, D** **)**.

In 57 patients with NSCLC, we further analyzed the prediction of survival based on biomarkers in patients receiving different PD-1 therapies. There was a statistically significant difference in PFS and OS in the clinically responsive/clinically non-responsive patients treated with chemotherapy combined nivolumab (P = 0.024, P = 0.048), as well as in the clinically beneficial and non-clinically beneficial groups (P = 0.024 and 0.048, respectively) (**Figures 5E, F** **)**. The other group received chemotherapy combined with pembrolizumab, and there was a significant difference in PFS and OS between the clinical response and non-clinical response cohort (P = 0.011, P = 0.037), whereas only PFS was statistically different between the clinical benefit and non-clinical benefit groups (P = 0.03) (**Figures 5G, H** **)**.

Combined with these results, IP10/IL8 (S2/S0) as a predictive marker can effectively predict the clinical efficacy and survival of patients. Both lung adenocarcinoma and lung squamous cell cancer and both chemotherapy combined with nivolumab and pembrolizumab had good predictive value. The lack of a statistical difference between individual groups might be caused by the small sample size. In future studies, we will include more samples for verification; as a predictor, there is no difference between them (**Supplemental Figures 2A, B** **)**.

## Discussion

Specific immunotherapy targeting cancer cells has been reported to be more attention than non-specific therapy methods, including surgery, radiotherapy, and chemotherapy (29). The PD-l/PD-L1 pathway has been suggested to play important roles in the escape mechanisms of tumor cells. Monoclonal antibodies for PD-1/PD-L1 blockade therapy, one of the major treatment strategies in cancer immunotherapy, are considered to significantly suppress tumor growth in multiple tumor models (30–32). Recently, increasing clinical evidence shows that chemo-combined immunotherapy is more effective than immunotherapy alone (33). Here, we propose a new biomarker for PD-1 blockade therapy to better monitor the outcomes of chemo-combined immunotherapy for patients with lung cancer and multiple/various other cancer types.

Cytokines comprise a category of small proteins that are important in cell signaling for growth, differentiation, and inflammatory or anti-inflammatory effects. Some cytokines have strong anti-tumor activity, interferon-alpha and interleukin-2 are used in the treatment of malignancies (34). Cytokines are produced by a variety of cells, including immune cells such as macrophages, B lymphocytes, T lymphocytes, and mast cells (35). Chemokines are an important part of cytokines, and they induce responsive cells to have directional chemotactic action. They are involved in a variety of immune and inflammatory reactions, such as recruiting activated T lymphocytes, neutrophils, monocytes, and natural killer cells from the blood to sites of infection or tissue damage and promoting wound healing via G protein-coupled receptors (36).

IP-10, also referred to as interferon γ-induced protein (CXCL10), is a 10 kDa secreted polypeptide belonging to the CXC chemokine family, which is involved in trafficking immune cells to inflammatory sites (37). IP10 is secreted by several cell types strongly in response to IFN-γ and IFN-α/β and weakly in response to TNFα. IP10 can also be induced by NF-κB and plays an early role in hypoxia-induced inflammation (38–40). In the last decade, IP10 was determined to be an immune response indicator. After therapeutic surgery, CXCL10 was found to be negatively associated with high levels of CD8 T cell infiltration, indicating poor tumor growth and recurrence (41). In an animal study, CXCL10-deficient mice infected with dengue virus presented with a higher mortality rate (42). A recent study also confirmed that IP10 can as a biomarker to predict clinical curative effects; the results show that levels of IP10 in peripheral plasma increased after treatment compared to baseline levels in clinically responsive patients. Patients who progressed after treatment had lower IP10 levels than those before treatment, and the baseline level of IP10 was determined to be associated with patient PFS (43). Macrophage-derived CXCL9 and CXCL10 are indispensable for the response of immune checkpoint inhibitors (anti-PD-1 and anti-CTLA-4), single-cell RNA-seq analysis of tumor-infiltrating lymphocytes (TIL) showed that CXCL9/CXCL10/CXCL11 was mainly expressed by macrophages following immune checkpoint blockade, this study showed that enhancing the production of CXCL9/CXCL10 to improve the efficacy of immunotherapy in patients, these findings can be used clinically for diagnosis or treatment (44).

Interleukin (IL) levels were found to be related to some types of advanced cancer. They are associated with a poor prognosis for malignant disease. Reitter et al. investigated the association between eight different cytokines and prognosis in patients with cancer. Higher IL-6, IL-8, and IL-11 levels were found to be associated with worse survival in patients with cancer, whereas elevated IL-10 and IL-6 levels might be markers of a favorable prognosis for colorectal cancer and lung cancer, respectively (45).

IL-8, a member of the CXC chemokine family, was originally thought to be a neutrophil-related chemokine (46). IL-8 has been found to be secreted under inflammatory stimulation of malignant cells and tumor stromal cells in different tumor types (47). Tumor invasiveness has been found to be mediated by the increased activation of matrix metalloproteinase-2 and -9 (MMP-2 and MMP-9), mediated by IL-8 levels, indicating that the IL-8 level is associated with metastatic invasiveness and early recurrence (48, 49). Sanmamed et al. found that the serum IL-8 level reflects tumor burden and acts as a biomarker to monitor therapeutic outcomes of vemurafenib and ipilimumab in patients with metastatic melanoma (50). Recent studies also revealed that the changes in serum IL-8 levels reflect clinical benefits from PD-1 blockade therapy in patients with melanoma and NSCLC. More importantly, they were also found to correctly discern responses in a small number of patients presenting with pseudoprogression, highlighting a potential role of serum IL-8 as a predictor of immunotherapy clinical outcomes (25).

In this study, 67 lung cancers were used as a discovery group and 24 different cancers were used as a validation group. We used the high-throughput cytokine detection platform and performed bioinformatics analysis of blood samples. This study is the first to propose that an evaluation of the cytokine IP10/IL-8 (S2/S0, ratio changes at 10–12 weeks after treatment) in peripheral blood can be a suitable biomarker with high specificity and sensitivity for tumor chemo-combined immunotherapy. The best cutoff value was then calculated based on the training set. The training set (lung cancer) and validation set (multiple cancers) were predicted, and this predictor could effectively distinguish the response and benefit after treatment. Moreover, there was a strong correlation between plasma IP10/IL-8(S2/S0) levels and patient survival. This would be useful to predict patients who will benefit from chemotherapy combined with immunotherapy. A more important result of our statistical algorithm is that the biomarker IP10/IL-8 (S2/S0) obtained in both CB/NCB and R/NR groups was the same. In addition, corresponding validation was also performed for different histological types of NSCLC and chemotherapy combined with different PD1 drug groups, and there was no difference between the histological types of lung cancer and different PD-1 antibodies in predicting the efficacy of this biomarker. We propose an innovative regression variable selection strategy that considers not only the goodness-of-fit but also the predictive ability based on the verification set and selects the optimal variables to improve the explanatory ability of the variables while optimizing the number of variables. This observation would play a critical role in the clinical guidance of patients using appropriate antibodies targeting the PD-1 signaling pathway and drug combinations.

## Conclusions

This study demonstrates the ratio of IP-10 to IL-8 (S2/S0, ratio of changes at 10-12 weeks after treatment to baseline) to predict the response to immunotherapy combined with chemotherapy and evaluate the survival of lung cancer patients and multiple cancer patients. Importantly, we show that for patients treated with combination therapy, the specificity and sensitivity of IL-8 and IP10 as predictors were better than those of IL-8 and IP10 alone, which was verified in not only lung cancer but also multiple cancer research cohorts. Moreover, the biomarker also showed a good predictive effect on different histological types of NSCLC and chemotherapy combined with different PD1 drug groups, it provides a new idea for the biomarkers of tumor combination therapy.

## Data Availability Statement

The original contributions presented in the study are included in the article/**Supplementary Material**. Further inquiries can be directed to the corresponding authors.

## Ethics Statement

Written informed consent was obtained from the individual(s) for the publication of any potentially identifiable images or data included in this article.

## Author Contributions

LLW, TL, and HW designed experiments. SX, LXW, JL, LH, BQ, QW, GZ, LZ, and WG collected patients’ samples and clinical information. LW and SJ analyzed data and designed the figures. LLW, SJ, and HW wrote the manuscript. All authors contributed to the article and approved the submitted version.

## Funding

This study was supported by National Natural Science Foundation of China (81672274) and National Science and Technology Major Project(2018ZX09201-013).

## Conflict of Interest

The authors declare that the research was conducted in the absence of any commercial or financial relationships that could be construed as a potential conflict of interest.

## Supplementary Material

Supplementary Figure 1 Cytokine combinations were used as biomarkers in multiple cancers to predict patient survival. **(A)** Kaplan–Meier plots of progression-free survival (PFS, left) and overall survival (OS, right) of LDA-R and LDA-NR based on cytokine combination I. **(B)** Kaplan–Meier plots of PFS (left) and OS (right) of LDA-R and LDA-NR based on cytokine combination II. **(C)** Kaplan–Meier plots of PFS (left) and OS (right) of LDA-CB and LDA-NCB based on cytokine combination I. **(D)** Kaplan–Meier plots of PFS (left) and OS (right) of LDA-CB and LDA-NCB based on cytokine combination II.

Supplementary Figure 2 IP10/IL8 (S2/S0) was used as a predictor in different histological types of NSCLC and different PD-1 drugs were analyzed. **(A)** Different histological types of lung cancer. **(B)** Chemotherapy combined with different PD-1 antibody drugs.
