# Supplementary figures and images for: The Ratio of IP10 to IL-8 in Plasma Reflects and Predicts the Response of Patients With Lung Cancer to Anti-PD-1 Immunotherapy Combined With Chemotherapy
Source: Front Immunol. 2021 Apr 12;12:665147. doi: 10.3389/fimmu.2021.665147 (PMC8072287; doi:10.3389/fimmu.2021.665147)

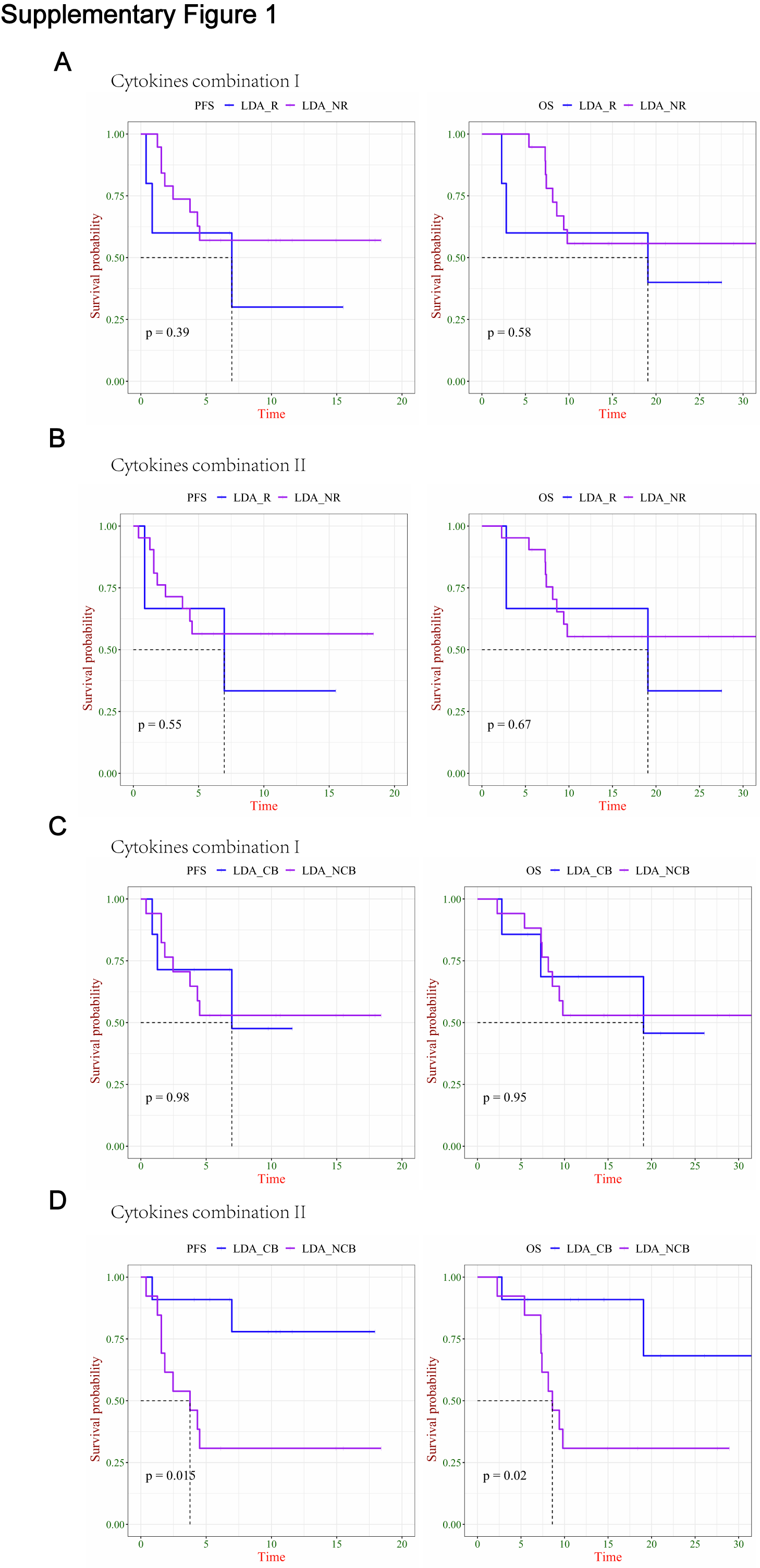

Supplement: Supplementary Figure 1 — Cytokine combinations were used as biomarkers in multiple cancers to predict patient survival. (A) Kaplan–Meier plots of progression-free survival (PFS, left) and overall survival (OS, right) of LDA-R and LDA-NR based on cytokine combination I. (B) Kaplan–Meier plots of PFS (left) and OS (right) of LDA-R and LDA-NR based on cytokine combination II. (C) Kaplan–Meier plots of PFS (left) and OS (right) of LDA-CB and LDA-NCB based on cytokine combination I. (D) Kaplan–Meier plots of PFS (left) and OS (right) of LDA-CB and LDA-NCB based on cytokine combination II. [file Image_1.tif]

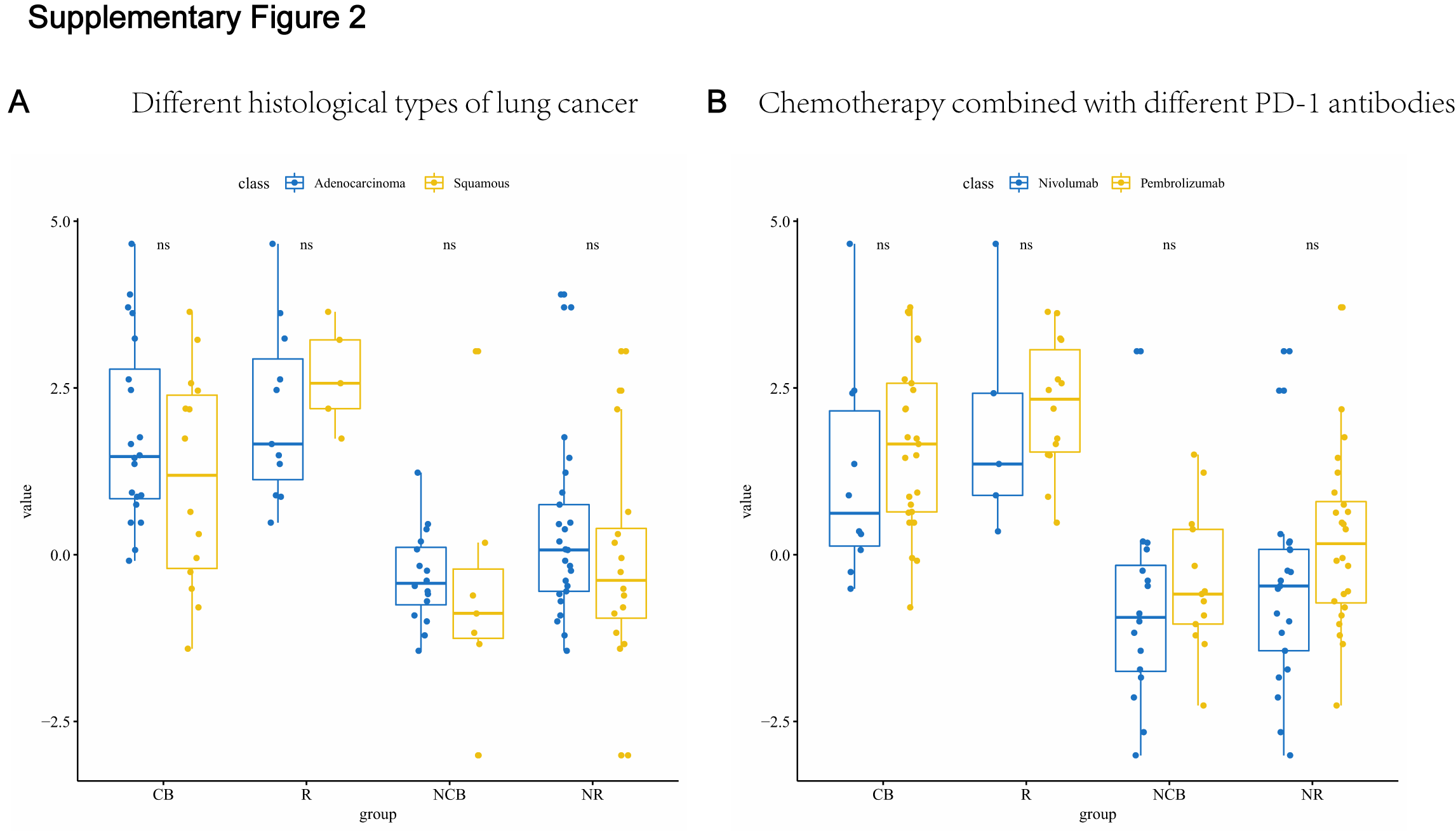

Supplement: Supplementary Figure 2 — IP10/IL8 (S2/S0) was used as a predictor in different histological types of NSCLC and different PD-1 drugs were analyzed. (A) Different histological types of lung cancer. (B) Chemotherapy combined with different PD-1 antibody drugs. [file Image_2.tif]
