# Supplementary material for: The Ratio of IP10 to IL-8 in Plasma Reflects and Predicts the Response of Patients With Lung Cancer to Anti-PD-1 Immunotherapy Combined With Chemotherapy
Source: Front Immunol. 2021 Apr 12;12:665147. doi: 10.3389/fimmu.2021.665147 (PMC8072287; doi:10.3389/fimmu.2021.665147)
Supplement: Supplementary file 3 [file DataSheet_1.pdf]

Supplemental Table 1 Factors with significant difference between R /NR and CB/NCB shown in Figure1

**Group**	**Time**	**Factor**	**Changes**	**P value**	**Fold change**	**AUC**
R/NR	S1	IL-2	Higher	0.0196	1.59	0.67
	S1	IP-10	Higher	0.0014	1.04	0.75
	S2	IL-8	Lower	0.0064	1.36	0.72
	S2	IL-10	Lower	0.0404	1.05	0.38
CB/NCB	S1	IL-7	Higher	0.0015	1.43	0.73
	S1	IL-8	Lower	0.0024	1.01	0.72
	S2	IL-8	Lower	0.00012	1.78	0.77
	S2	IL-6	Lower	0.0039	1.93	0.71
	S2	IL-2	Higher	0.0274	1.14	0.65
	S2/S0	IL-4	Lower	0.0483	1.11	0.64

P value for distinction between R/NR and CB/NCB(Wilcoxon' s rank-sum test). The area under the curve (AUC) of each cytokines in relation to responsiveness was calculated from univariate logistic regression analysis.

Supplementary Table 2 Cytokines showing a significant difference in responders (R) and non-responders (NR), clinical benefit (CB) and no clinical benefit (NCB)

Group	Time	Cytokine	P-value
**R/NR**	S0	IL-1ra	0.033264
	S1	IP-10	0.00141
		IL-2	0.019552
	S2	IP-10	0.003808
		IL-8	0.006398
		IL-10	0.04045
	S1/S0	IP-10	0.000519
	S2/S0	IL-8	0.000015
		IL-1ra	0.008389
		MIP-1a	0.009844
		IP-10	0.015802
		G-CSF	0.041563
	S2/S1	IL-8	0.002847
		MCP-1(MCAF)	0.032531
		RANTES	0.037496
**CB/NCB**	S1	IL-7	0.001489
		IL-8	0.002374
		MIP-1b	0.011464
		G-CSF	0.014423
		IL-5	0.029176
	S2	IL-8	0.000123
		IL-6	0.003926
		IL-2	0.027361
		IL-13	0.039939
	S2/S0	IL-8	0.000033
		IP-10	0.001804
		MIP-1a	0.003645
		Eotaxin	0.029628
		IL-4	0.048361
	S2/S1	IL-8	0.019339
		IP-10	0.035784
		Eotaxin	0.03691

Supplementary Table 3 The best predictive Factor selected by AIC regression procedure in R/NR group

Factor Combination	Selected Factor	Changes in R to NR	Sample
I	IP-10 (S1/S0)	Higher	S0+S1
	IL-2 (S1)	Higher	
	IP-10 (S1)	Higher	
II	IL-8 (S2/S0)	Lower	S0+S1+S2
	IP-10 (S1)	Higher	
	MCP-1(MCAF) (S2/S1)	Lower	
	IP-10 (S2/S0)	Higher	
	RANTES (S2/S1)	Higher	
	IL-2(S1)	Higher	

Supplementary Table 4 The best predictive Factor selected by AIC regression procedure in CB/NCB group

Factor Combination	Selected Factor	Changes in CB to NCB	Sample
I	IL-7 (S1)	Higher	S0+S1
	IL-8 (S1)	Lower	
	G-CSF (S1)	Higher	
II	IL-8 (S2)	Lower	S0+S1+S2
	IP-10 (S2/S0)	Higher	
	IL-7 (S1)	Higher	
	IL-8 (S2/S0)	Lower	
	IL-8 (S2/S1)	Higher	
	Eotaxin (S2/S0)	Higher	
	IL-13 (S2)	Higher	

Supplementary Table 5 Logistic regression models.

Group	Logistic Regression Model
CB/NCB	Z=1.2387* $IP10^{log\frac{S2}{S0}}$-1.2029* $IL8^{log\frac{S2}{S0}}$ -0.1882
R/NR	Z=0.9414* $IP10^{log\frac{S2}{S0}}$ -1.0717* $IL8^{log\frac{S2}{S0}}$ -1.9317

$IP10^{log\frac{S2}{S0}}$ is $\log_2 (IP10^{s2}/IP10^{s0})$, $IL8^{log\frac{S2}{S0}}$ is $\log_2 (IL8^{s2}/IL8^{s0})$

Supplementary Table 6 The process of the logistic regression models transformation.

Group	Model Transformation Process
CB/NCB	$Z = -0.1882 + \log_2\left(\frac{\left(IP10^{\frac{s2}{s0}}\right)^{1.2387}}{\left(IL8^{\frac{s2}{s0}}\right)^{1.2029}}\right)$ $\approx -0.1882 + \log_2\left(\frac{IP10^{\frac{s2}{s0}}}{IL8^{\frac{s2}{s0}}}\right)^{1.2}$ $= -0.1882 + 1.2 * \log_2\left(\frac{IP10^{\frac{s2}{s0}}}{IL8^{\frac{s2}{s0}}}\right)$
R/NR	$Z = -1.9317 + \log_2\left(\frac{\left(IP10^{\frac{s2}{s0}}\right)^{0.9414}}{\left(IL8^{\frac{s2}{s0}}\right)^{1.0717}}\right)$ $\approx -1.9317 + \log_2\left(\frac{IP10^{\frac{s2}{s0}}}{IL8^{\frac{s2}{s0}}}\right)^{1.0}$ $= -1.9317 + 1.0 * \log_2\left(\frac{IP10^{\frac{s2}{s0}}}{IL8^{\frac{s2}{s0}}}\right)$
